# Accelerating AI innovation in healthcare: real-world clinical research applications on the Mayo Clinic Platform

**DOI:** 10.1038/s44401-026-00068-1

**Published:** 2026-02-16

**Authors:** Yue Yu, Xinyue Hu, Sivaraman Rajaganapathy, Jingna Feng, Ahmed Abdelhameed, Xiaodi Li, Jianfu Li, Xiaoke Liu, Liu Yang, Nilüfer Ertekin-Taner, Phil Fiero, Soulmaz Boroumand, Richard Larsen, Maneesh Goyal, Clark C. Otley, Nansu Zong, Vijay H. Shah, John D. Halamka, Cui Tao

**Affiliations:** 1 Department of Artificial Intelligence and Informatics, Mayo Clinic, Jacksonville, FL USA; 2 Department of Cardiovascular Medicine, Mayo Clinic Health System, La Crosse, WI USA; 3 Division of Hepatology and Liver Transplant, Mayo Clinic, Jacksonville, FL USA; 4 Department of Neurology, Mayo Clinic, Jacksonville, FL USA; 5 Department of Neuroscience, Mayo Clinic, Jacksonville, FL USA; 6 Mayo Clinic Platform, Rochester, MN USA; 7 Department of Dermatology, Mayo Clinic, Rochester, MN USA; 8 Division of Gastroenterology and Hepatology, Mayo Clinic, Rochester, MN USA

**Keywords:** Business and industry, Computational biology and bioinformatics, Health care, Medical research, Scientific community

## Abstract

Artificial intelligence (AI) holds promise for healthcare, but real-world implementation remains difficult. The Mayo clinic platform (MCP) addresses this by providing scalable, multi-institutional, de-identified data and analytical tools. Through four research projects, we demonstrate MCP’s ability to support efficient cohort identification, AI model development, and real-world evidence generation. MCP enables broader accessibility and standardization compared to institutional EHRs, positioning it as a powerful platform for advancing translational research and precision medicine.

## Introduction

In recent years, artificial intelligence (AI) has emerged as a transformative force poised to revolutionize the field of biomedicine^1,2^. However, the transition of AI algorithms from in silico simulations to practical, real-world clinical applications remains challenging^3,4^. Effective implementation of AI in healthcare requires a comprehensive integration of the entire ecosystem, extending beyond the algorithms themselves^5^. For instance, within the medical domain, a prominent trend is the development of multimodal AI models that integrate diverse data types across multiple modalities^6–8^. This advancement, however, introduces complex issues, such as safeguarding patient privacy amidst the aggregation of sensitive information^9,10^. From the perspective of AI model development, advancing beyond retrospective design and validation poses an additional hurdle^11,12^. Moreover, ensuring the accessibility of advanced tools and adequate computational resources to accommodate the variable requirements of individual users is essential for widespread adoption and effectiveness^9,13^. Another significant challenge is the integration of expert-in-the-loop systems that require no-code AI platforms^14^, which are crucial for enabling non-technical medical professionals to effectively use and interact with AI tools without needing extensive programming knowledge.

To accelerate the development of medical AI, several established initiatives, including i2b2/TranSMART^15,16^ and OHDSI/OMOP^17,18^, have significantly advanced real-world data integration and analytics. In addition, large-scale research platforms such as the All of Us Research Program^19,20^ and the UK Biobank^21,22^, have emerged to support AI research and data science studies. These platforms offer standardized longitudinal real-world data—both All of Us and UK Biobank offer EHR data in the OMOP common data model (CDM) format and secure, cloud-based environments designed for high-performance computing.

Since 2019, Mayo Clinic started to create the Mayo clinic platform (MCP)^23^, which focuses on transforming healthcare through data science and digital health technologies. By leveraging a vast array of standardized clinical data, advanced analytics, and collaborative networks like the Mayo Clinic Care Network, the platform aims to improve patient care and streamline health outcomes. It fosters innovation by enabling healthcare organizations, providers, and digital health companies to access real-time insights and deploy cutting-edge solutions.

**Fig. 1:**
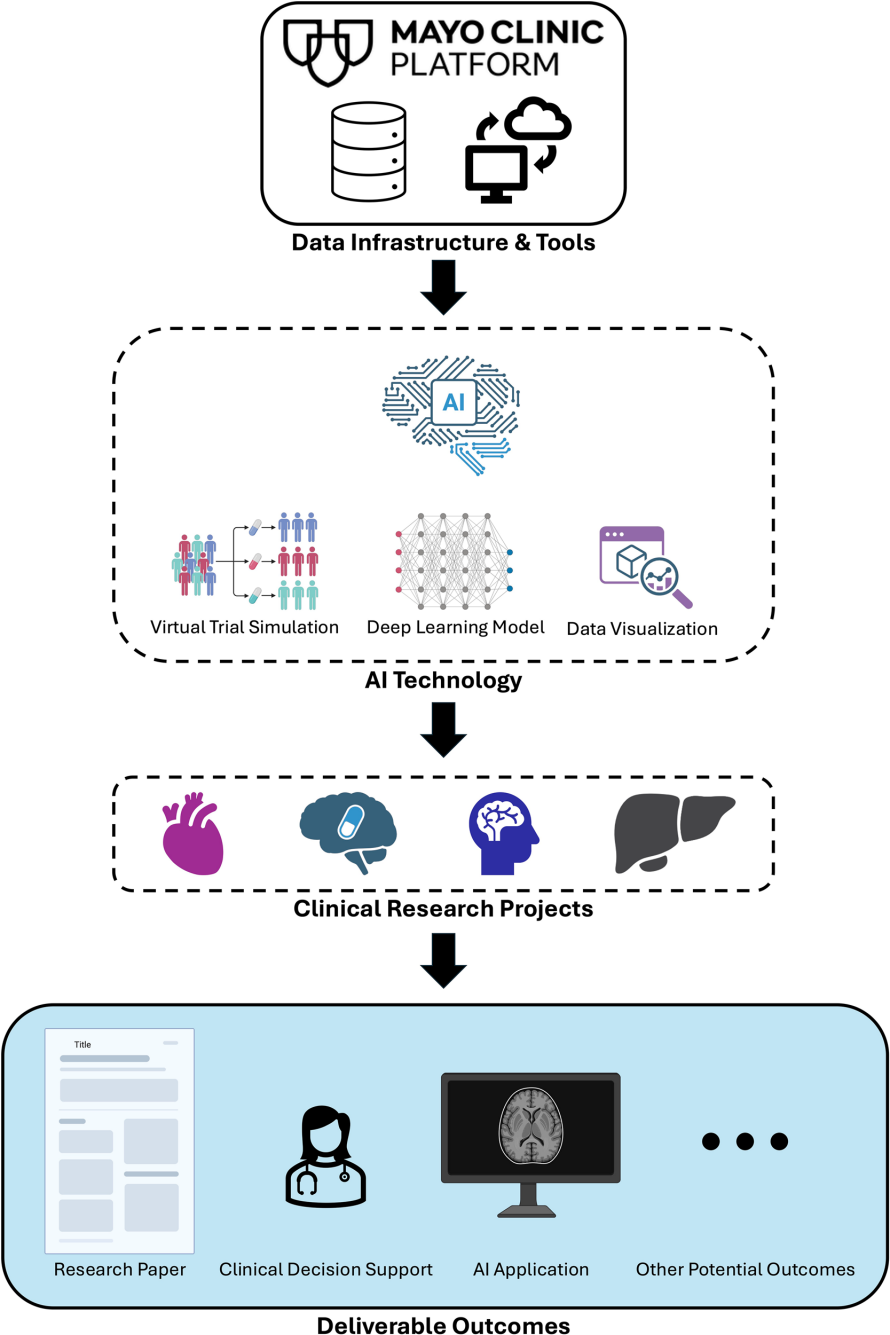
Workflow of the four demonstration projects. This figure summarizes the workflow and four research projects conducted on MCP and the analytical or AI technologies used in each. **Project 1:** RCT emulation for heart failure drug efficacy using AI simulation frameworks. **Project 2:** RCT emulation and evaluation of antihypertensive medications and dementia risk. **Project 3:** Prediction of MCI-to-AD progression with a BiGRU deep learning model. **Project 4:** Prediction of post-transplant MACE using neural network–based deep learning models.

In this brief communication, we aim to demonstrate how MCP enables real-world clinical research and AI innovation through practical applications. Specifically, we explore the platform’s capabilities by conducting four representative research projects using real-world EHR data and integrated MCP tools (Fig. 1). By leveraging MCP’s robust data infrastructure and versatile toolset—from intuitive visualizers to advanced AI workspaces—these projects collectively showcase how MCP facilitates scalable, reproducible, and collaborative research. Rather than providing exhaustive technical details for each project, this study highlights the platform’s integrative features, including standardized multi-institutional data, privacy-preserving design, and accessible analytical environments. Together, these examples demonstrate MCP’s pivotal role as an enabling infrastructure for AI-driven clinical research, accelerating translational medicine and advancing precision healthcare.

## Demonstration Projects Enabled by MCP

Table 1 summarizes four clinical research projects conducted using the MCP, which includes both standard statistical analysis and AI-based research. For the randomized controlled trial (RCT) stimulation project, the Cohort Visualizer tool was used to build the study cohort. All projects also utilized the Schema Visualizer and Workspaces for EHR data collection and analysis. The results highlight the effectiveness of MCP’s data, tools, and computing environment in facilitating successful data science research. MCP contributed to significant outcomes across all projects, including the development of a reusable research pipeline, scientific validation of existing studies, and AI-based prediction model. Specifically, Project 1 delivered a reusable pipeline for stimulating RCTs using real-world data, offering a cost-effective alternative for evaluating treatment efficacy. Project 2 validated prior findings through robust statistical analysis, contributing real-world evidence to support antihypertensive medications in reducing dementia risk. Project 3 validated a deep learning model for predicting MCI-to-AD progression via the EHR data from diverse healthcare systems, showcasing the potential of AI in early disease detection. Project 4 created an advanced predictive model for major adverse cardiovascular events (MACE) following liver transplantation, enabling improved clinical risk stratification. These outcomes highlight MCP’s capacity to accelerate data-driven research, support translational science, and generate actionable insights that can enhance patient care.

**Table 1:** Overview of Clinical Research Projects Leveraging MCP

Project	Project 1:RCT Stimulating for HF Drug Efficacy	Project 2:AHMs Impact on ADRD	Project 3:MCI-to-AD Progression Prediction	Project 4:MACE after LT prediction
**Research Method**	Simulation Framework	Statistical Analysis	Deep Learning	Deep Learning
**MCP Tools Involved**	Cohort Visualizer,Schema Visualizer,Workspaces	Schema Visualizer,Workspaces	Schema Visualizer,Workspaces	Schema Visualizer,Workspaces
**MCP Data Involved**	Structured EHR data(demographic, diagnosis, procedure, lab test, medication)	Structured EHR data(demographic, diagnosis, procedure, lab test, medication)	Structured EHR data(demographic, diagnosis, procedure, medication)	Structured EHR data(demographic, diagnosis, procedure, medication)
**Deliverable Outcomes by using MCP**	(1) Pipeline for stimulation of drug efficacy RCTs, (2) Potential research paper	(1) Scientific validation for existing study, (2) Potential research paper	(1) AI model for MCI-to-AD prediction, (2) Potential research paper	(1) AI model for MACE after LT prediction, (2) Potential research paper

As an example of system performance, the MACE after LT prediction project illustrates MCP’s efficiency. For a researcher familiar with the MCP data structure (or OMOP CDM), it typically takes about one week to collect all required structured EHR data (demographics, diagnoses, procedures, and medications) for approximately 15,000 patients. Using a medium-computing configuration (6 CPU cores, 38 GB RAM, no GPU), it takes only about 10 min to train and run the BiGRU deep learning model. This demonstrates that MCP can support both large-scale data processing and rapid model development, providing an efficient and accessible environment for real-world machine learning research.

## Key contributions of MCP for real-world AI research

In this study, we demonstrated that MCP has played a critical role in enabling clinical studies using real-world EHR data. While multiple platforms have enabled advances in AI research, this paper focuses on the MCP environment to illustrate practical workflows, collaborations, and outcomes. The MCP provides not only comprehensive, standardized, de-identified, and multiple institutional real-world data, but also powerful tools in the data science and healthcare domains. We appreciated key features such as the Cohort Visualizer, Schema Visualizer, and Workspaces. Our studies not only yielded publishable results from the research perspective, but also effectively leveraged AI-driven methodologies to address real-world clinical challenges, reinforcing the platform’s impact on both academic research and clinical innovation.

While multiple data-sharing and analytics frameworks—such as i2b2/TranSMART and OHDSI/OMOP—have provided valuable infrastructure and tools for real-world evidence research, MCP extends these concepts by integrating federated, multi-institutional data with standardized OMOP CDM formatting and embedding comprehensive research tools within a single cloud-based environment. This approach not only ensures interoperability with existing data standards but also expands accessibility for external researchers through a subscription-based model, supporting both open-source and proprietary analytic pipelines. By combining secure de-identified data access, code-free interfaces, and AI-ready computing environments, MCP serves as a next-generation platform that bridges real-world data analytics and AI-driven translational medicine. Its hybrid design enables scalability and reproducibility while ensuring privacy and compliance. This synthesis of data standardization, federated architecture, and integrated AI development distinguishes MCP as a novel, comprehensive framework for accelerating healthcare innovation.

Compared to traditional institutional real-world data research repositories—that is, standardized real-world clinical data repositories created within individual institutions for research use—the MCP offers distinct advantages for clinical research (as Table 2 shows). MCP provides de-identified data, streamlining IRB approvals and accelerating research timelines for users. Additionally, it enables external researchers to access high-quality Mayo EHR data for study analysis and validation, whereas institutional research repositories are usually restricted to internal use^24^. MCP also incorporates extensive data standardization, particularly for unstructured clinical notes, by offering AI-powered processing to synthesize standardized data representations, thereby improving the utility of unstructured text for clinical decision sopport^25^. In contrast, most of the institutional research repositories primarily rely on medical billing codes as the main data record for research use. Furthermore, MCP is more than a data warehouse—it supports a broad range of users through integrated tools that facilitate research across skill levels, from code-free interfaces to advanced programming environments, enhancing accessibility. In comparison, using institutional repositories is more coding-intensive, requiring a steeper learning curve^26^. Moreover, MCP will not only integrate Mayo Clinic’s data but also data from other academic medical centers who partner with the MCP to contribute de-identified data to MCP’s federated data network (each, a “Data Network Partner”), thereby broadening the scope of available research data. By offering these capabilities, MCP enhances data analysis, improves model validation, and facilitates more efficient and reliable clinical research.

**Table 2:** Advantages of MCP Compared to the Institutional Real-World Data Research Repositories

Feature	MCP	Institutional Research Repositories
Data Access	Available for both Mayo Clinic and external researchers	Restricted to internal use
Data Type	De-identified data	Identifiable data
Data Standardization	Extensive standardization	Limited standardization
Tool Accessibility	Supports both code-free and coding-dependent tools	More coding-intensive
Learning Curve	Lower, accommodates various user skill levels	Higher, due to coding dependency
Data Integration	Integrates Mayo data and data from other Data Network Partners	Internal data within the institution

To improve accessibility, MCP provides both no-code and code-enabled tools to support researchers with diverse technical backgrounds. The Cohort Visualizer and Schema Visualizer allow non-technical users to explore data and define cohorts through intuitive interfaces, while advanced users can utilize Workspaces and coding environments such as JupyterLab and RStudio for customized analyses. We recognize that accessibility for users with limited data science or machine learning expertise remains a continual area for enhancement. Ongoing development efforts aim to further expand low-code and guided-analytics features, enabling clinicians and other domain experts to engage in AI-driven research more effectively. These initiatives align with MCP’s broader vision to democratize data science and make AI-powered research more inclusive across the healthcare community.

A limitation of this study is that all four projects focused exclusively on structured EHR data within MCP, without incorporating other data modalities. For example, MCP also supports the processing and analysis of unstructured EHR data, including free-text clinical notes, through integrated natural language processing (NLP) and large language model (LLM) pipelines. In the future, we plan to use additional data types of MCP, including clinical notes, medical images, and omics data, to broaden research opportunities. Furthermore, as external datasets become available, cross-validation across institutions will further strengthen clinical research. Additionally, MCP provides state-of-the-art AI deployment capabilities via infrastructure designed to streamline the integration of AI solutions into clinical workflows. While we have yet to implement these four research projects using such deployment capabilities, future studies will explore its capabilities to facilitate AI deployment and assess its potential to accelerate the translation of AI-driven innovations into real-world clinical practice. By leveraging MCP, we aim to bridge the gap between research and clinical application.

In the era of AI, the MCP is poised to revolutionize clinical research by advancing multimodal AI, real-world evidence generation, and global data collaboration. By integrating structured EHR data, clinical notes, imaging, and genomics, researchers can leverage MCP harmonized data to enhance biomedical knowledge for large medical foundation models. This integration will boost downstream tasks such as predictive analytics for early disease detection and personalized treatment^7,8^. MCP also ensures robust and generalizable AI model validation across multiple institutions. Its data ecosystem will facilitate large-scale studies while maintaining patient privacy, effectively bridging the gap between AI research and real-world clinical implementation^27^. Moreover, MCP may transform drug development by enabling real-world evidence-based trials that extend beyond traditional clinical settings. This approach allows for broader participation and more diverse data collection, enhancing trial efficiency and relevance^28^. In addition, MCP can facilitate our “Clinical Trials Beyond Walls” approach which allows broader participation by removing barriers for patient involvement and includes initiatives with underserved communities to enhance the relevance and quality of clinical trials^29^. With scalable research tools and expanded accessibility, MCP will empower a diverse research community, accelerating medical innovation and driving the future of precision medicine and proactive healthcare.

## Method

### Platform Architecture Overview

The MCP is a secure, cloud-based data science environment designed to accelerate research and innovation through access to large-scale, de-identified, standardized clinical data and integrated analytical tools. The platform architecture is built to ensure scalability, privacy, and accessibility for researchers across diverse disciplines.

Extensive De-identified and Standardized Data Resources: The MCP employs an innovative de-identification and standardization process applied to data from more than 15.1 million patients. To safeguard patient privacy, the platform uses a multilayered de-identification strategy the combines rule-based heuristics and deep learning models to identify and replace personally identifiable information^30^. These measures ensure full compliance with HIPAA and institutional governance policies. In addition, the platform provides extensive data standardization, including mapping EHR data to standard medical terminologies and common data models. This rich, multimodal dataset enables a wide range of research applications, including AI model training, real-world evidence generation, and clinical insight discovery.

Integrated Research Tools: The MCP provides a comprehensive suite of research tools that streamline the entire data-to-discovery workflow. These tools enable secure data access, exploration, and analytics within a unified platform. Designed for scalability and ease of use, the MCP tool ecosystem supports both technical and non-technical users, promoting efficient, reproducible, and collaborative research across diverse data types while maintaining rigorous standards for privacy, governance, and compliance.

Dedicated Data Science Environment: Researchers access MCP through a secure, cloud-hosted data science environment tailored for their use. This environment integrates the MCP research tools and provides preconfigured support for open-source analytical frameworks such as Python, R, and TensorFlow. It offers controlled, compliant access to de-identified data and high-performance computing resources, enabling seamless model training and evaluation within a managed and privacy-preserving infrastructure.

This architecture establishes MCP as a scalable, privacy-preserving, and AI-ready research environment that enables investigators to generate actionable insights from de-identified real-world data while maintaining the highest standards of security and compliance.

### Real-world observational data in MCP

MCP provides access to extensive, high-quality clinical data, including standardized structured data (e.g., diagnoses, lab results, medications) and unstructured data (e.g., clinical notes, images). This de-identified data spans diverse demographics and captures patient journeys over time. Currently, MCP’s datasets include over 15.1 million patient records, 12 billion radiology images, 3.2 billion lab results, and 1.65 billion clinical notes, all accessible through a secure data science environment. In addition to the Mayo specific standardized EHR, MCP also provides EHR data in the OMOP CDM format, which enhances interoperability and allows users to leverage analytic pipelines and tools developed within the OHDSI ecosystem.

### MCP tools used in this study

MCP partners with nference, inc^31^. to make available various tools to accommodate different needs. In this study, since we only used structured EHR data within MCP, the following tools were utilized.

**Fig. 2:**
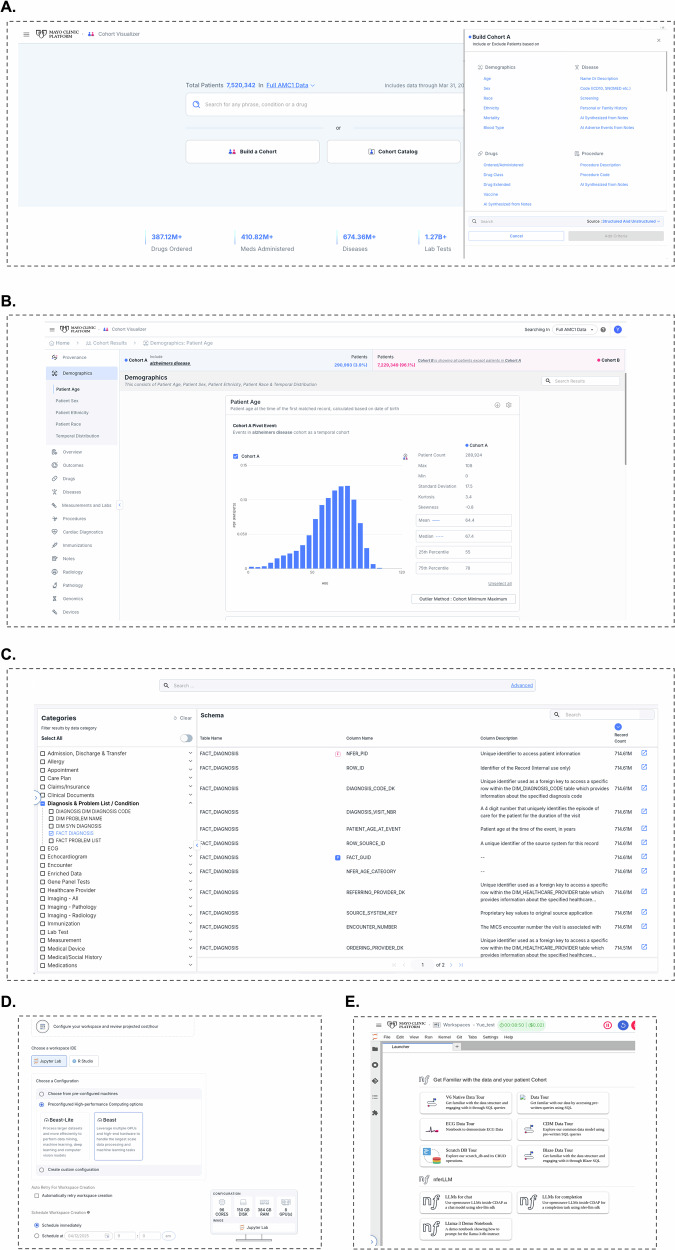
Interface of the MCP Tools. **A**, **B** Interface of the Cohort Visualizer, showing patient cohort creation **A** and comparison views **B**. **C** Interface of the Schema Visualizer, illustrating exploration of the data schema and relationships between tables. **D**, **E** Interface of the MCP Workspace, demonstrating coding environments (**D**, e.g., JupyterLab and RStudio) and integrated computational tools for data analysis and AI model development **E**.

Cohort Visualizer facilitates the quick creation, characterization, and comparison of patient cohorts for hypothesis testing and analysis using EHR data. It supports both structured and unstructured data, offering code-free analytics and intuitive visualization tools. Users can load or create new cohorts and analyze them using graphical or tabular formats by the cohort builder. With user-friendly navigation, it allows users, regardless of technical expertise, to explore vast clinical datasets using standard clinical codes or keywords, helping to accelerate clinical research and address unmet needs in translational medicine. Additionally, for more detailed downstream analysis, it provides SQL code to facilitate data retrieval from the EHR database. Figure 2A shows the user interface of the MCP Cohort Builder, where users can define and filter patient cohorts using structured/unstructured EHR data. Figure 2B illustrates the Cohort Comparison interface, which allows users to visualize and compare cohort characteristics through graphical summaries.

Schema Visualizer provides an interactive interface for exploring the data dictionary and schema within MCP. It offers detailed information on tables, columns, and their relationships, along with query code examples for downstream data collection (Fig. 2C). Additionally, it features an advanced search tool that enables users to efficiently locate specific tables, columns, or values within the data schema.

Workspaces in MCP offer a comprehensive environment for accessing data and computing resources, supporting advanced analytics and data science workflows. The platform provides scalable computational resources tailored to a variety of research needs. For an individual researcher, the maximum available configuration includes 208 CPU cores, 1872 GB of RAM, and 8 NVIDIA H100 80 GB GPUs, ensuring capacity for complex, data-intensive machine learning workflows. They also provide the latest open-source tools, packages, and libraries for cloud-based computation, with integrated support for JupyterLab, VSCode, and RStudio to accommodate diverse coding needs. This all-in-one platform streamlines data collection, processing, and analysis. Additionally, Workspaces include high-performance computing capabilities for resource-intensive tasks such as data mining, machine learning, and deep learning. They also offer code-level guidance for various applications, including data extraction, large language model (LLM) execution, and medical image processing. Furthermore, users can leverage Git within Workspaces to efficiently manage and collaborate on their repositories in GitHub. Figure 2D, E shows the interface page of the MCP workspace.

### Research projects conducted on MCP

**Fig. 3:**
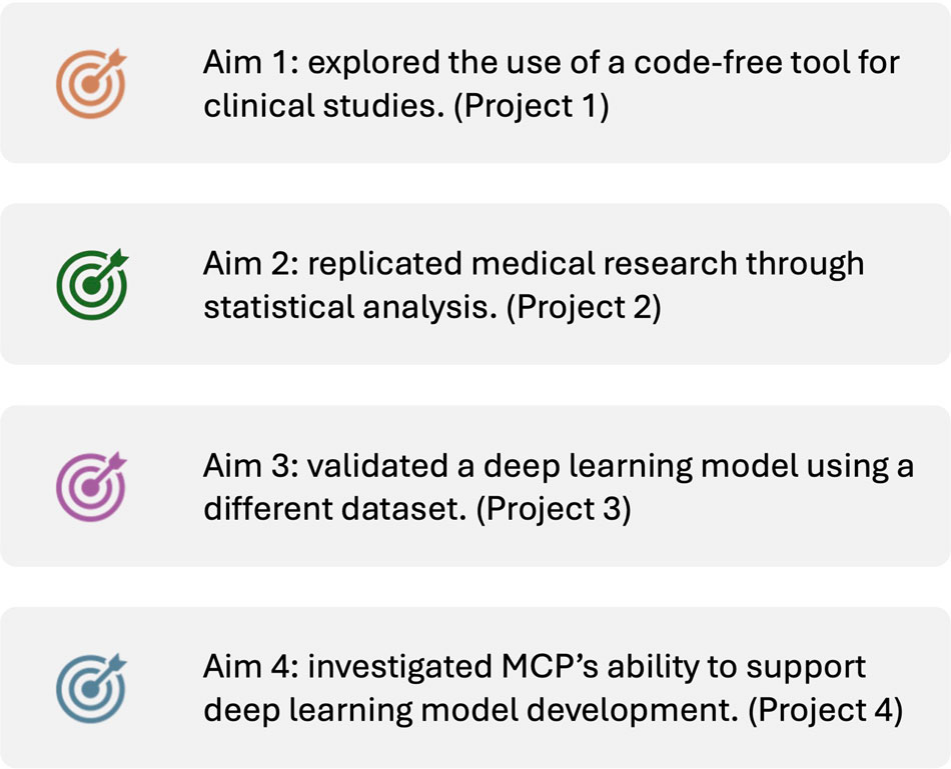
Aims of the Four Clinical Research Projects.

To comprehensively showcase MCP’s capabilities across various clinical research scenarios, we designed four distinct projects. Figure 3 illustrates the aims of these projects within their respective clinical research contexts. Detailed descriptions of each project are provided below.

Project 1. Stimulating drug efficacy randomized controlled trials (RCTs) for heart failure (HF) patients using real-world observational clinical data. This project leverages the rich retrospective data available on MCP to stimulate the conditions of traditional randomized controlled trials (RCTs). By doing so, it enables high-quality research that sidesteps the usual costs and ethical concerns associated with traditional RCTs. More specifically, we developed methodologies to stimulate RCTs for evaluating drug efficacy in HF patients using real-world observational data. Key objectives include identifying suitable RCT candidates for stimulation and leveraging EHR data to replicate heart failure drug efficacy trials, thereby enabling robust comparative effectiveness research in the absence of traditional RCTs. Additionally, this project explores the use of the Cohort Visualizer, a code-free analytical tool designed for researchers without a data science background, facilitating accessible and efficient cohort analysis.

Project 2. Impact of antihypertensive medications (AHMs) on Alzheimer’s Disease and Related Dementias (ADRD) risk in hypertensive patients with mild cognitive impairment (MCI). This study aims to validate findings from a prior study^32^ that suggested AHM use may be associated with a reduced risk of ADRD in hypertensive patients with MCI. Utilizing real-world observational data, the primary objective is to perform survival analysis to assess the relationship between AHM use and ADRD progression. Additionally, the study investigates potential drug-drug interactions between AHMs, statins, and metformin within the target patient cohort, providing further insights into pharmacological influences on dementia risk. This project serves as a simulation of traditional clinical research, employing statistical analysis to assess real-world evidence.

Project 3. Building a Mild Cognitive Impairment (MCI) to Alzheimer’s Disease (AD) progression prediction model using EHR data and deep learning method. This project focuses on training and validating a deep learning model^33^ to predict the progression from MCI, considered to be a prodrome to dementia^34^, to AD using longitudinal EHR data. Specifically, it employs the Bidirectional Gated Recurrent Units (BiGRU) deep learning model to forecast MCI progression at varying time intervals, extending up to five years post-diagnosis. Additionally, the study aims to validate the model’s generalizability across diverse datasets and healthcare systems, ensuring its applicability in real-world clinical settings.

Project 4. Developing Deep Learning Model to predict Major Adverse Cardiovascular Events (MACE) After Liver Transplantation (LT). This project focuses on leveraging longitudinal EHR data to develop advanced deep learning models on the MCP for predicting MACE following LT and to compare the performance with our previously developed model based on medical claims data^35^. By identifying high-risk candidates, the model aids clinicians in risk stratification and informs management strategies to improve transplant outcomes. Additionally, the model highlights key predictive features, enabling physicians to implement targeted preventive measures to reduce the likelihood of adverse cardiovascular events. This study demonstrates the capability of MCP in facilitating deep learning model development for clinical research.

### Data collection and analysis approach

The MCP tools have played a crucial role in facilitating these projects by providing a unified platform for cohort development, data extraction, and analysis. Specifically, Project 1 leveraged the Cohort Visualizer to identify RCT candidates. Subsequently, all projects utilized Jupyter Notebook to execute SparkSQL API queries for extracting EHR data from the MCP database. Finally, data analysis—including statistical evaluations and deep learning modeling—was conducted within the Workspace using either R or Python.

### Platform accessibility and reusability

The MCP is a subscription-based, cloud-hosted research environment accessible to external users following registration and approval. Researchers, healthcare organizations, and industry partners can register to access MCP’s de-identified datasets and integrated tools by completing the required onboarding process. Once registered, users have access to the same standardized data, analytical tools, and secure computing environments described in this paper. The platform supports both open-source and proprietary components—users can utilize open-source tools (e.g., Python, R, TensorFlow, PyTorch) within the MCP Workspaces, ensuring flexibility and reproducibility. This hybrid model promotes collaboration, scalability, and replicable research while maintaining robust privacy and security protections.

## Data Availability

This study involves analysis of de-identified Electronic Health Record (EHR) data via the Mayo Clinic Platform. Data shown and reported in this manuscript has been extracted from the EHR using an established protocol for data extraction, aimed at preserving patient privacy. The data has been determined to be de-identified pursuant to an expert’s evaluation, in accordance with the HIPAA Privacy Rule. Any data beyond what is reported in the manuscript, including but not limited to the raw EHR data, cannot be shared or released due to the parameters of the expert determination to maintain the data de-identification. Contact corresponding authors for additional details regarding the Mayo Clinic Platform.
